# A Compositional Analysis of Physical Activity, Sedentary Time, and Sleep and Associated Health Outcomes in Children and Adults with Cystic Fibrosis

**DOI:** 10.3390/ijerph19095155

**Published:** 2022-04-23

**Authors:** Mayara S. Bianchim, Melitta A. McNarry, Anne Holland, Narelle S. Cox, Julianna Dreger, Alan R. Barker, Craig A. Williams, Sarah Denford, Kelly A. Mackintosh

**Affiliations:** 1 Applied Sports, Technology, Exercise and Medicine Research Centre, Swansea University Bay Campus, Swansea SA1 8EN, UK; tuca_may@hotmail.com (M.S.B.); k.mackintosh@swansea.ac.uk (K.A.M.); 2 Nursing, Midwifery and Allied Health Professions Research Unit, University of Stirling, Stirling FK9 4LA, UK; 3 Department of Allergy, Immunology and Respiratory Medicine, Monash University, Melbourne 3004, Australia; anne.holland@monash.edu (A.H.); narelle.cox@monash.edu (N.S.C.); jdreger@ucalgary.ca (J.D.); 4 Alfred Health, Australia Institute for Breathing and Sleep, Melbourne 3004, Australia; 5 Alfred Health, Physiotherapy Department, Melbourne 3004, Australia; 6 Children’s Health and Exercise Research Centre, University of Exeter, Exeter EX1 2LU, UK; a.r.barker@exeter.ac.uk (A.R.B.); c.a.williams@exeter.ac.uk (C.A.W.); sarah.denford@bristol.ac.uk (S.D.); 7 Population Health Sciences, Bristol Medical School, University of Bristol, Bristol BS8 1UD, UK

**Keywords:** movement behaviours, moderate-to-vigorous physical activity, lung function, composition, youth, compositional analysis

## Abstract

This study sought to investigate the association of light physical activity (LPA), moderate-to-vigorous physical activity (MVPA), sedentary time (SED), and sleep with lung function in children and adults with CF. In total, 86 children (41 females; 13.6 ± 2.8 years; FEV_1_%_predicted_: 86 ± 1%) and 43 adults (21 females; 24.6 ± 4.7 years; FEV_1_%_predicted_: 63 ± 21%) with CF participated in this study. Wrist-worn accelerometery was used to assess PA, SED and sleep. Compositional linear regression models were conducted following normalisation via isometric log-ratio transformations. Sequential binary partitioning was applied to investigate the impact of reallocating 10 to 30 min between each behaviour on FEV_1_%_predicted_. A decline in FEV_1_%_predicted_ was predicted with the reallocation of 30 min from MVPA to SED or LPA or sleep to any other behaviour in children (−3.04–−0.005%) and adults (−3.58–−0.005%). Conversely, improvements in FEV_1_%_predicted_ were predicted when 30 min was reallocated to MVPA from LPA or SED in children (0.12–1.59%) and adults (0.77–2.10%), or when 30 min was reallocated to sleep from any other behaviour in both children (0.23–2.56%) and adults (1.08–3.58%). This study supports the importance of MVPA and sleep for maintaining and promoting lung function in people with CF.

## 1. Introduction

Cystic fibrosis (CF) is the most prevalent life-shortening inherited disorder affecting over 10,500 people in the United Kingdom (UK) [1]. Regular physical activity (PA) is an important component of CF care and is associated with multiple benefits [2], including better quality of life and prolonged life expectancy [3]. Despite that, research investigating the association between PA and health outcomes in people with CF has reported equivocal findings [4,5,6,7]. There is discrepancy regarding which PA intensity is associated with lung function in children with CF [6,8,9]. Similarly, in adults with CF, whilst Cox, et al. [5] and Savi, et al. [4] found that moderate-to-vigorous physical activity (MVPA) was associated with better lung function, such an association was not found by Savi, et al. [8]. Furthermore, other studies have shown that higher self-reported PA levels are positively associated with a slower decline in lung function in both children and adults with CF [7,9]. Such discrepancies may be due, at least in part, to the failure to account for the compositional nature of PA.

PA behaviours are highly collinear [10,11], with each intensity representing a proportion of the total waking time [12]. Thus, the amount of time allocated to one behaviour will directly affect the time available for all others [13]. PA research has increasingly recognised the value of integrating all movement behaviours, including sleep and sedentary time (SED), through the use of compositional analyses [12,13,14]. Compositional data analysis accounts for the collinearity of PA data by expressing the relative information as a set of log ratios [13], which can be transposed from the compositional sample space (*d*-simplex), and analysed with traditional models (i.e., linear regression). The use of compositional analysis has enabled researchers to explore the effects of increasing MVPA at the expense of SED in adults, on reducing mortality risk [15], cardiometabolic biomarkers [16] and diabetes [17]. Compositional analyses of PA data in CF can identify to the amount of time that needs to be displaced from each behaviour in order to elicit beneficial health outcomes and can be important for informing the design of successful PA interventions. However, to date, no research has utilised compositional analysis to ascertain the relationship between PA and the primary health outcome for those with CF, forced expiratory volume in the first second predicted (FEV_1_%_predicted_).

The aim of this cross-sectional study was to use compositional analysis to investigate the association between time spent asleep, sedentary and in LPA, MVPA and FEV_1_%_predicted_ in children and adults with CF.

## 2. Materials and Methods

### 2.1. Participants

In total, 104 children and 43 adults with CF were recruited for this study, with adulthood being defined as 18+ years. Amongst these, 42% (*n* = 62) were homozygous for ΔF508 mutation [p.Phe508del (c.1521_1523delCTT)] and 25% (*n* = 22) had Cystic Fibrosis-Related Diabetes (CFRD). Participants were recruited from Paediatric CF Clinics in South Wales and from adult CF clinics in Australia. Those with multi-resistant bacteria (e.g., Cepacia and Nontuberculous (NTM)), co-morbidities that might compromise being physically active (e.g., cardiovascular and musculoskeletal) or who were awaiting a transplant were excluded from this study. Prior to study commencement, all participants provided written informed consent, and parent/guardian assent was obtained for all participants under 18 years of age. Ethics approval was obtained from the National Health Service (NHS) Research Ethics Committee (18/WS/0032) in the United Kingdom and from the Human Research Ethics Committee at Alfred Health in Australia (HREC/16/Alfred/188).

### 2.2. Measurements

Body mass and stature were measured to the nearest 0.1 kg and 0.1 cm, respectively, and body mass index (BMI) was subsequently calculated and presented as z-scores for children and adolescents. A standard spirometry assessment using a forced vital capacity manoeuvre was performed to determine lung function [18]. Spirometry was assessed in accordance with American Thoracic Society and European Respiratory Society standards [19]. FEV_1_ was obtained, and FEV_1_%_predicted_ estimated, using a reference equation [20], and subsequently utilised to indicate disease severity as mild (>70%_predicted_), moderate (40–69%_predicted_) or severe <40%_predicted;_ [21]. Genotype and the presence of CFRD were extracted from the medical records.

Habitual PA was assessed by two different accelerometers, ActiGraph GT9X Link (*n* = 43 adults and 53 children; ActiGraph, Pensacola, FL) and GENEActiv (*n* = 33 children; ActivInsights Ltd., Cambridge, UK), secured on the non-dominant wrist for seven consecutive days. Participants were instructed to wear the monitors at all times. Raw accelerometer data were extracted at 100 Hz as .gt3x and .bin files using ActiLife V 6.10.2 and GENEActiv PC software V2.2, respectively. All .gt3x files were converted to time-stamp free .csv files and then imported, along with the .bin files, into R statistical software (V3.1.2; R Foundation for Statistical Computing, Vienna, Austria). The GGIR package (V1.2–0; http://cran.r-project.org, accessed on 3 June 2019) [22] was used to auto-calibrate the data, detect abnormal values, and detect non-wear time. Subsequently, the Euclidean Norm Minus One (ENMO) was determined from the vector magnitude by subtracting one gravitational unit from the three raw acceleration signals at each time-stamp. Only those with at least four days and three nights of valid accelerometer data, with ≥16 h of wear-time in each day, were included in the final analyses [23]. The procedure utilised to detect non-wear time is described in detail elsewhere [24]. Hildebrand, et al. age- and brand-specific cut-points were used to estimate time accumulated in sedentary, LPA and MVPA in 5 s epochs [25].

Sleep was assessed using a validated algorithm integrated within the GGIR package [26,27]. Briefly, the algorithm detects sleep time as any period of sustained inactivity, defined as no change of more than five degrees in the monitor angle during a nocturnal sleep window [26]. For the present study, the nocturnal window was identified using the heuristic algorithm developed by van Hees, et al. [27] for wrist-worn accelerometers. Finally, all data were visually inspected to ensure that this was in accordance with the nocturnal sleep pattern for population of similar ages [26].

### 2.3. Statistical Analysis

Descriptive statistics (mean and standard deviation (SD)) and frequencies were calculated for continuous and categorical variables, respectively, with an independent t-test used to compare groups (IBM SPSS Statistics; Version 23.0; IBM Corp., Armonk, NY, USA). Significance was accepted at an alpha of ≤0.05. Compositional analysis was performed in R using the ‘compositions’ and ‘robCompositions’ packages [13]. Initially, all datasets were screened to ensure that no zero values would be included in the composition. Subsequently, the compositional mean of each behaviour (sleep, SED, LPA, and MVPA) was computed across an average of all valid days. Then, a variation matrix was calculated for logs of all possible pair-wise ratios between the movement behaviours, with all pairs achieving values close to zero considered as presenting high proportionality. The relative data including all movement behaviours were presented as isometric log-ratio (*ilr*) coordinates [28,29].

Multiple linear regression was used, with the *ilr* co-ordinates as the explanatory variables, to explore the relationship between each behaviour and FEV_1_%_predicted_ [14]. Following a crude model with no adjustment for confounders, a subsequent model was run accounting for age, sex and genotype. All covariates were selected according to clinical relevance and relevance to PA levels [30].

To determine how the reallocation of time between behaviours impacts FEV_1_%_predicted_, the difference between each predicted outcome at the reference composition and at a new composition was estimated as described by Dumuid, et al. [14]. The reference composition comprised the averages of all behaviours linearly scaled to add to one (i.e., one day, 24 h in terms of the behaviour data), and the reallocation of time between different behaviours from the reference constituted the new composition, i.e., LPA to MVPA [14]. Lastly, binary partitioning was applied to estimate how the reallocation of time from each behaviour, in relation to the reference composition, impacted FEV_1_%_predicted_ [14,31]. This approach was repeated until all possible combinations of behaviours were analysed, with time reallocations increasing from 10 to 30 min in 10 min increments [12,13,14,31]. Ternary plots displaying the relationship between all movement behaviours were created to allow the visualisation of the *d*-simplex.

## 3. Results

Following the exclusion of 18 participants who did not meet the wear-time criteria, 86 children (41 girls; 13.6 ± 2.8 years) and 43 adults (21 females; 24.6 ± 4.7 years) were included in the analyses. There were no significant differences in anthropometric characteristics or lung function between those included and excluded from the analysis (*p* > 0.05). Descriptive characteristics and lung function data are presented in Table 1. Children and males heterozygous for DF508 had significantly higher FEV_1_%_predicted_ (*p* < 0.05) in comparison to adult males and all adults, respectively. Seventy-one participants had mild lung disease (57 children), 53 had moderate lung disease (21 children) and a minority had severe lung disease (1 child and 4 adults). Adults had a higher BMI (*p* = 0.007) and lower FEV_1_%_predicted_ (*p* = 0.004) than children, with children demonstrating lower MVPA (*p* < 0.0001) and longer sleep times (*p* = 0.04) than their adult counterparts. Irrespective of age, females demonstrated a lower FEV1. In addition, girls accumulated less MVPA than boys (*p* = 0.02); and females accrued less LPA (*p* = 0.04) and more SED (*p* = 0.05) than males.

The standard and compositional means describing the accumulation of time spent in each behaviour, along with the variation matrix of movement, are shown in Table 2 for children and adults. The results from the variation matrix indicate that, irrespective of age, sleep and LPA were the most highly (proportional) co-dependent, whilst SED and sleep represented the least co-dependent pair.

The unadjusted and adjusted linear regression models including the *ilr* coordinates as explanatory variables to predict lung function are presented in Table 3. In children, all non-adjusted models resulted in a multiple R^2^ of 0.12, an adjusted R^2^ of 0.08 and a *p*-value of 0.002; and all adjusted models resulted in a multiple R^2^ of 0.18, an adjusted R^2^ of 0.11 and a *p*-value of 0.02. In adults, all non-adjusted models resulted in a multiple R^2^ of 0.07, an adjusted R^2^ of −0.02 and a *p*-value of 0.53; and all adjusted models resulted in a multiple R^2^ of 0.20, an adjusted R^2^ of 0.02 and a *p*-value of 0.38. In the adjusted models, none of the covariates were significant in children or adults. In children, sleep was positively associated with all the remaining behaviours, whilst the model designed to retain sleep as *ilr* coordinate was negatively associated with all the other behaviours. No significant associations were found in adults.

### Sequential Binary Partitioning

The estimated changes in FEV_1_%_predicted_ with the non-adjusted model for each 10, 20 and 30 min of time reallocated between behaviours are shown in Table 4. In children, all reallocations from sleep to other behaviours (i.e., SED, LPA, and MVPA) resulted in a reduction in estimated lung function, whilst time displaced from all movement behaviours to sleep increased the estimated lung function. Conversely, in adults, reallocation from sleep to all other behaviours resulted in increases in estimated lung function. In particular, time displaced from sleep to MVPA resulted in a 2.24% increase in FEV_1_%_predicted_. Further, in adults, time displaced from LPA to SED or MVPA resulted in a reduced predicted lung function. Time displaced from SED to all other behaviours increased predicted lung function, with the exception of SED displaced to sleep in adults. Finally, time displaced from MVPA to LPA elicited a marginal increase in lung function in adults.

The estimated changes in FEV_1_%_predicted_ when reallocating 30 min amongst the behaviours, stratified by age, sex and genotype, are presented in Table 5. Similar to the non-stratified analysis, all displacements from sleep to other behaviours resulted in reductions in the estimated FEV_1_%_predicted_, whereas all reallocations from any behaviour to sleep resulted in an increase in estimated FEV_1_%_predicted_. LPA displaced to MVPA resulted in an increased FEV_1_%_predicted_ whilst the displacement of LPA to SED resulted in reduced FEV_1_%_predicted_ in children and adults. Further, stratifying groups according to age, sex and genotype revealed that time reallocated from MVPA to SED and LPA reduced lung function, irrespective of age, genotype or sex.

**Figure 1:**
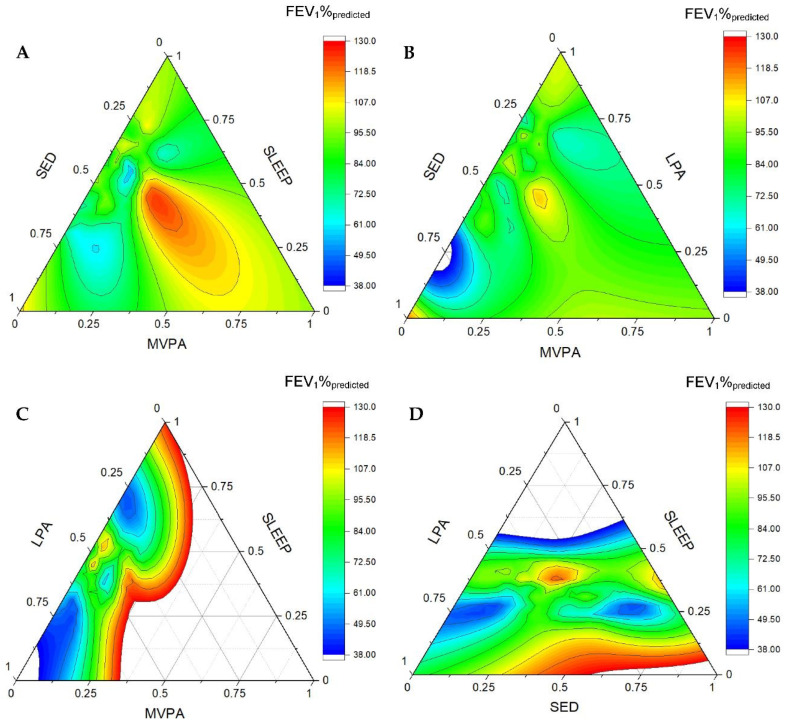
Ternary plots displaying the association between movement behaviours and FEV_1_%_predicted_ in children with cystic fibrosis. Each plot displays the relationship between three behaviours. The heat map represents the distribution of data points, with difference in colours indicating changes in FEV_1_%_predicted__._ (**A**) Sedentary behaviour, sleep and moderate-to-vigorous activity. (**B**) Sedentary behaviour, light physical activity and moderate-to-vigorous physical activity. (**C**) Light physical activity, sleep and moderate-to-vigorous physical activity. (**D**) Light physical activity, sleep and sedentary behaviour. SED: sedentary time, LPA: light physical activity, MVPA: moderate-to-vigorous physical activity, and FEV_1_%_predicted__:_ forced expiratory volume in the first second predicted.

**Figure 2:**
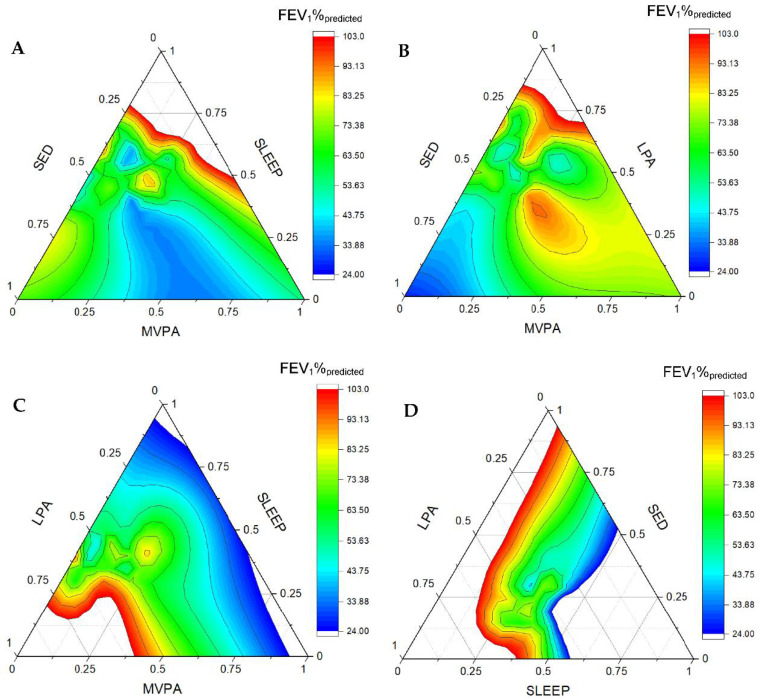
Ternary plots displaying the association between movement behaviours and FEV_1_%_predicted_ in adults with cystic fibrosis. Each plot displays the relationship between three behaviours. The heat map represents the distribution of data points, with difference in colours indicating changes in FEV_1_%_predicted_. (**A**) Sedentary behaviour, sleep and moderate-to-vigorous activity. (**B**) Sedentary behaviour, light physical activity and moderate-to-vigorous physical activity. (**C**) Light physical activity, sleep and moderate-to-vigorous physical activity. (**D**) Light physical activity, sleep and sedentary behaviour. SED: sedentary time, LPA: light physical activity, MVPA: moderate-to-vigorous physical activity, and FEV_1_%_predicted_ forced expiratory volume in the first second predicted.

Figure 1 and Figure 2 are ternary diagrams displaying the association between movement behaviours and FEV_1_%_predicted_ in children and adults, respectively. Each plot displays the relationship between three behaviours. The heat map represents the distribution of data points, with difference in colours indicating changes in FEV_1_%_predicted__._ The accumulation of MVPA and sleep favoured FEV_1_%_predicted_ in children and adults. In addition, the accumulation of LPA and SED is associated with lower FEV_1_%_predicted_ in children, whereas only more time spent in SED was associated with reduced lung function in adults.

## 4. Discussion

This study sought to investigate the association between time spent in sleep, SED, LPA, MVPA and FEV_1_%_predicted_ in children and adults with CF. Overall, irrespective of age, SED and LPA demonstrated the greatest co-dependency, which is in accordance with research in the healthy population [12,32,33]. This indicates that individuals who accumulated high amounts of SED also accrued a high proportion of time in LPA. More time in MVPA and sleep are associated with better FEV_1_%_predicted_, regardless of age. Increasing sleep, at expense of any behaviour, and MVPA, at expense of LPA, are strategies that might benefit lung function.

The present study found that the reallocation of 30 min from LPA and SED to MVPA, and from all movement behaviours to sleep, estimated a percentage predicted change in FEV_1_%_predicted_ of up to 2.56% in children and 3.58% in adults. It is noteworthy that the clinical significance of a change in FEV_1_ might vary according to disease severity, with more severe cases benefiting greatly from even small improvements. Nonetheless, given that FEV_1_%_predicted_ declines from 1.0 to 3.1% per year in children and adults with CF [34,35], achieving an up to 2.56–3.58% increase in FEV_1_%_predicted_ from the reallocation of different movement behaviours may have substantial clinical benefit. In addition, the magnitude of change in FEV_1_%_predicted_ with increasing MVPA and sleep, seen in the present analysis for the adjusted models, is akin to the percentage changes reported in studies evaluating the effect of medications for maintenance of lung health in CF [36,37,38]. For example, results from a large randomised trial including children and adults with CF indicated that the mean absolute improvement in the percentage of FEV_1_%_predicted_ ranged from 2.6 to 4.0% with the use of a CFTR corrector (lumacaftor) in combination with a potentiator (ivacaftor) [37]. The present study, therefore, contributes to the emerging literature showing that an integrated approach to PA promotion and behaviour change can be more beneficial than emphasis on isolated PA behaviours [39].

Congruent with earlier studies investigating different health markers [12,14,39], the present study demonstrated that prioritising sleep, in comparison to SED, LPA and MVPA was associated with the best predicted outcome. More specifically, the present study found that even a 30 min reduction in sleep was associated with detrimental effects on lung function in both children (−1.03–−2.46) and adults (−1.41–−3.58%). A recent systematic review and meta-analysis reported a direct correlation between FEV_1_%_predicted_ and fragmentation of sleep in children and adults with CF, suggesting such disturbances were also associated with an increased frequency of exacerbation and a deterioration in nutritional status and quality of life [40]. Most importantly, Barbosa, et al. [41] found that sleep disorders and nocturnal hypoxemia are prevalent in children and adolescents with CF, and are associated with worse clinical score and higher morbidity in this population. Sleep disorders are associated with exercise intolerance and increased SED in children with CF [41]. Additionally, research utilising non-compositional physical activity data analysis reported that sleep fragmentation is related to reduced MVPA in adults with CF [42]. Similarly, habitual PA, particularly at higher intensities, is associated with longer sleep in children and adults with CF [43]. These findings suggest that whilst sleep and PA seem to influence each other, they also mutually affect lung function, and disease progression. As such, future studies developing PA recommendations or interventions in people with CF are strongly advised to account for sleep, in line with the Canadian 24 h movement guidelines [44].

Research investigating the relationship between PA and clinical outcomes in CF remains sparse and has mainly focused on individual movement behaviours [45]. Nonetheless, longitudinal investigations have reported a slower decline in FEV_1_ with regular PA in paediatric [9] and adult cohorts [7,10]. Amongst these studies, the only one using accelerometery to measure PA showed that accumulating 30 min of MVPA daily is associated with slower FEV_1_ decline in adults with CF [10]. However, whilst MVPA is well recognised as fundamental to health promotion and maintenance, particularly in those with CF, evidence regarding the association between lung function and PA remains controversial [11,46]. Specifically, Cox, et al. [5] found that adults with CF that accumulated more than 30 min of MVPA per day had better lung function than their peers. In contrast, Savi, et al. [8] found no associations between FEV_1_%_predicted_ and MVPA in adults with CF. Furthermore, Mackintosh, et al. [6] found that LPA, but not MVPA, was related to FEV_1_ in children with CF. Most importantly, the present study demonstrates that MVPA resulted in enhanced estimates of lung function when the time was reallocated only from LPA and SED, and not sleep.

An important consideration is that lung function predictions, resulting from reallocating the composition, were asymmetrical for all movement behaviours, with the exception of sleep (except for girls heterozygous for DF508). Previous studies utilising compositional analysis have also found asymmetrical relationships between movement behaviours and other outcomes in healthy [33,47,48] and pre-diabetic [17] populations. Essentially, asymmetry is observed when the alteration in a certain movement behaviour does not predict the exact same magnitude of change with the reverse reallocation. For example, in the present study, reduced MVPA is associated with a greater magnitude of deleterious change in FEV_1_%_predicted_ in both children (−0.005–−2.11%) and adults (−0.005–−2.22%) than to the estimated benefit following the proportional increase in MVPA. This finding has important clinical implications given that FEV_1_%_predicted_ is related to survival in CF [49]. Therefore, encouraging the maintenance of daily MVPA while reducing SED should be paramount in clinical service delivery, irrespective of age, sex or genotype.

Despite previous research showing that LPA is related with reduced inflammatory markers [32], there is still a lack of research investigating the clinical benefits of LPA in people with CF. Mackintosh et al. [6] reported that LPA was related to FEV_1_ in children with CF. In agreement, the present study demonstrated that the reallocation of 30 min of SED to LPA resulted in improved estimations of lung function in both children and adults with CF. This finding holds important clinical implications particularly regarding delaying lung function decline and slowing disease progression, even if those improvements were modest in comparison to the benefits associated with increasing sleep or MVPA. Specifically, those with CF spend less time in MVPA in relation to their healthy peers [50,51], which has been attributed to exercise intolerance associated with the condition [52]. Therefore, large reallocations of time to LPA from SED are particularly important in moderate and severe cases of the condition, which are characterised by exercise intolerance and muscle weakness [51].

This study demonstrated that the displacement of SED resulted in improved estimates of FEV_1_%_predicted_, regardless of the behaviour being reallocated to. Specifically, the reallocation of 30 min of SED to sleep resulted in the greatest increase in FEV_1_%_predicted_ in both children (1.87–2.56%) and adults (3.20–3.58%), whilst the reallocation to MVPA resulted in the lowest, also in children (−2.11–−1.47%) and adults (−2.22–−1.94%). Surprisingly, the displacement of 30 min from SED to LPA resulted in marginally greater estimates of FEV_1_%_predicted_ in children (0.35–0.98%) and adults (1.13–1.37%) in comparison to the same amount of time reallocated to MVPA. Nonetheless, it is notable that the reduction in SED might have meaningful implications for people with CF. For example, Polito, et al. [53] reported that adults with CF who spent longer being sedentary had an increase in inflammatory markers, in comparison to those who engaged in more PA. In addition, SED is broadly recognised as a major risk factor for disease and is associated with metabolic markers in healthy children [54] and adults [55]. This is especially relevant given that SED increases with age [56], in parallel with the complexity of the exercise intolerance and airway disease due to the progressive nature of CF [57]. Finally, the use of compositional analysis indicated that while increasing MVPA appears to be one of the most optimal stimuli to enhance lung function, this should not be achieved as the expense of sleep. However, in scenarios where it is not possible to increase MVPA, due to disease severity for example, large increments in LPA with time reallocated from SED may provide similar benefit to FEV_1_%_predicted_.

Overall, this study was associated with numerous strengths, not least the use of compositional analysis using device-measured SED and PA whilst accounting for factors such as age, sex and genotype. The utilisation of cut-points from raw metrics, as opposed to count-based cut-points, is critical given that count-based thresholds have been associated with low accuracy and high error [58]. Finally, a large sample was utilised, including a broad range of age and disease severity.

Regardless of the strengths, some limitations need to be considered. First, this study utilised a cross-sectional approach, and therefore, causality cannot be established. As such, any changes observed in FEV_1_%_predicted_ arising from the reallocation of each behaviour warrant careful interpretation and future longitudinal research is warranted to confirm these findings. In addition, two different monitor brands were utilised in this study, which might generate some variability in the PA estimations. Moreover, whilst CF-specific cut-points for children and adolescents were recently developed [59], similar condition-specific thresholds are not currently available for adults. Therefore, age- and accelerometery brand-specific cut-points [25,60] were utilised for children and adults in order to maintain consistency across age groups, despite the potential bias associated with this approach. Another important consideration is regarding the heterogeneity of the sample that included participants across the spectrum of age and disease severity (including outpatients and inpatients) from two different countries. Whilst this heterogeneity might help to generalise the study findings, it also might affect the estimation of PA. Lastly, it is noteworthy that the participants included in the present study presented relatively high levels of MVPA, which might have impacted the results, and consequently, the generalisability of these findings to less physically active people with CF.

## 5. Conclusions

This is the first study to use compositional analysis to investigate the impact of reallocating different movement behaviours on lung function in children and adults with CF. Estimated improvements in FEV_1_%_predicted_ were associated with reallocating 30 min from SED to sleep, and from LPA to MVPA, in both children and adults. Importantly, these results were predicted irrespective of age, sex and genotype. Overall, these findings reinforce the importance of accounting for the full spectrum of movement behaviours, and are imperative to inform future studies tailoring PA interventions regarding the amount of time and the direction of the reallocation warranted to enhance lung function for those with CF. Clinical teams are encouraged to continue to promote MVPA and good sleep habits to impact future lung health.

## Figures and Tables

**Table 1:** Descriptive characteristics, physical activity levels and lung function for children and adults by sex.

	Children			Adults		
Variable (Unit)	Overall (*n* = 86)	Girls (*n* = 41)	Boys (*n* = 45)	Overall (*n* = 43)	Females (*n* = 21)	Males (*n* = 22)
Age (years)	13.6 ± 2.8	13.7 ± 2.7	13.5 ± 2.8	24.6 ± 4.7	23.6 ± 3.5	25.5 ± 5.5
Height (cm)	154.2 ± 14.9 ^1^	152.5 ± 13.1	155. ± 16.13	166.0 ± 28.7	153.2 ± 35.6 ^2^	178.1 ± 10.0
BMI (kg·m^−2^)	18.7 ± 3.4 ^1^	18.8 ± 2.5	18.6 ± 4.1	21.2 ± 4.4	20.0 ± 5.3	22.4 ± 2.9
zBMI	−0.2 ± 0.9	−0.1 ± 0.8	−0.3 ± 1.1	-	-	-
FEV1 (l)	2.3 ± 0.8	2.1 ± 0.7 ^2^	2.5 ± 0. 7	2.4 ± 1.0	1.9 ± 0.7 ^2^	2.9 ± 1.0
FEV_1_%_predicted_ (%)	86 ± 21 ^1^	84 ± 24	88 ± 18	63 ± 21	62 ± 1	64 ± 21
Sleep (min)	479.3 ± 70.6 ^1^	471.2 ± 63.4	487.0 ± 77.5	453. 3 ± 64.5	452.1 ± 68.0	454.5 ± 62.6
SED (min)	344.8 ± 165.7	382.1 ± 196.0	313.7 ± 123.1	341.5 ± 116.0	381.3 ± 122.9 ^2^	303 ± 97.1
LPA (min)	562.0 ± 140.5	543.7 ± 167.9	574.6 ± 105.5	529.6 ± 121.1	491.3 ± 125.7 ^2^	566.3 ± 106.8
MVPA (min)	53.7 ± 115.3 ^1^	42.8 ± 32.0 ^2^	64.5 ± 53.0	115.3 ± 83.6	115.1 ± 68.6	115.5 ± 97.5

Data are presented as the mean ± SD. FEV1: forced expiratory volume in one second, FEV_1_%_predicted_: forced expiratory volume in the first second predicted, BMI: body mass index. ^1^ Significant difference between children and adults (*p* ≤ 0.05). ^2^ Significant difference between sex within the age groups (*p* ≤ 0.05).

**Table 2:** Unadjusted and compositional means and variation matrix of movement behaviours in children and adults with CF.

	Sleep	SED	LPA	MVPA
*Children*				
**Unadjusted (min)**	477.2 ± 74.2	345.0 ± 166.5	561.7 ± 136.3	56.0 ± 50.3
**Compositional**	0.34	0.22	0.39	0.02
**Sleep**	__	0.05	0.02	−0.15
**SED**	0.05	__	−0.05	−0.34
**LPA**	0.02	−0.05	__	−0.11
**MVPA**	−0.15	−0.34	−0.11	__
*Adults*				
**Unadjusted (min)**	457.9 ± 61.8	331.1 ± 98.9	528.8 ± 114.3	122.0 ± 84.2
**Compositional**	0.33	0.23	0.37	0.06
**Sleep**	__	0.06	0.03	−0.18
**SED**	0.06	__	0.02	−0.25
**LPA**	0.03	0.02	__	−0.16
**MVPA**	−1.18	−0.25	−0.16	__

Mean ± SD. SED: sedentary time, LPA: light physical activity, and MVPA: moderate-to-vigorous physical activity.

**Table 3:** Unadjusted and adjusted linear regression models for FEV_1_%_predicted_ in children and adults with CF.

		Children						Adults					
		Non-Adjusted			Adjusted			Non-Adjusted			Adjusted		
Retained *ilr* Coordinate	Clinical Covariates	Regression Coefficient	Standard Error	*p*-Value	Regression Coefficient	Standard Error	*p*-Value	Regression Coefficient	Standard Error	*p*-Value	Regression Coefficient	Standard Error	*p*-Value
	Sleep	13.22	4.46	0.004 *	12.77	4.80	0.009 *	−7.68	9.61	0.43	−16.66	10.64	0.13
	SED	−2.85	1.65	0.08	−2.97	1.77	0.09	−4.27	4.93	0.39	2.04	6.63	0.76
MVPA	LPA	−3.56	2.18	0.10	−3.04	2.26	0.18	4.40	4.39	0.32	3.19	4.85	0.51
	Sex	N/A	N/A	N/A	6.86	5.08	0.18	N/A	N/A	N/A	10.27	11.01	0.35
	Age	N/A	N/A	N/A	−1.35	0.77	0.08	N/A	N/A	N/A	−1.52	0.99	0.13
	Genotype	N/A	N/A	N/A	2.46	5.15	0.63	N/A	N/A	N/A	14.49	8.83	0.11
	SED	−7.09	2.30	0.002 *	−7.06	2.49	0.006 *	−1.46	6.50	0.82	7.48	8.59	0.39
	LPA	−8.84	3.57	0.01 *	−8.15	3.75	0.03 *	8.15	7.33	0.27	10.28	7.55	0.18
Sleep	MVPA	−8.19	3.05	0.009 *	−8.05	3.32	0.01 *	5.30	6.32	0.40	11.41	7.07	0.11
	Sex	N/A	N/A	N/A	6.86	5.08	0.18	N/A	N/A	N/A	10.27	11.01	0.35
	Age	N/A	N/A	N/A	−1.35	0.77	0.08	N/A	N/A	N/A	−1.52	0.99	0.13
	Genotype	N/A	N/A	N/A	2.46	5.15	0.63	N/A	N/A	N/A	14.49	8.83	0.11
	LPA	−5.97	3.12	0.05	−5.33	3.26	0.10	8.17	6.49	0.21	7.19	6.99	0.31
	MVPA	−2.27	1.59	0.15	−2.28	1.73	0.19	3.92	2.92	0.19	4.64	2.93	0.12
SED	Sleep	12.44	3.89	0.002 *	12.15	4.20	0.005 *	−3.80	9.23	0.68	−14.78	11.10	0.19
	Sex	N/A	N/A	N/A	6.86	5.08	0.08	N/A	N/A	N/A	10.27	11.01	0.35
	Age	N/A	N/A	N/A	−1.35	0.77	0.08	N/A	N/A	N/A	−1.52	0.99	0.13
	Genotype	N/A	N/A	N/A	2.46	5.15	0.63	N/A	N/A	N/A	14.49	8.83	0.11
	MVPA	−0.15	1.27	0.90	−0.37	1.36	0.78	0.97	2.12	0.64	1.97	2.26	0.39
	Sleep	13.97	4.61	0.003 *	13.41	4.94	0.008 *	−7.80	10.04	0.44	−16.97	11.07	0.13
LPA	SED	−0.68	2.11	0.74	−1.05	2.23	0.63	−5.90	5.53	0.29	0.17	7.2	0.98
	Sex	N/A	N/A	N/A	6.86	5.08	0.08	N/A	N/A	N/A	10.27	11.01	0.35
	Age	N/A	N/A	N/A	−1.35	0.77	0.08	N/A	N/A	N/A	−1.52	0.99	0.13
	Genotype	N/A	N/A	N/A	2.46	5.15	0.63	N/A	N/A	N/A	14.49	8.83	0.11

FEV_1_%_predicted_: forced expiratory volume in the first second predicted, SED: sedentary time, LPA: light physical activity, and MVPA: moderate-to-vigorous physical activity, N/A: non-applicable. * Statistical significance (*p* ≤ 0.05).

**Table 4:** Changes in FEV_1_%_predicted_ when reallocating time amongst different movement behaviours from the non-adjusted model in children with CF.

	Children			Adults		
**Reallocation**	*10 min*	*20 min*	*30 min*	*10 min*	*20 min*	*30 min*
Sleep to SED	−1.14 (−1.61–−0.73)	−2.27 (−3.27–−1.41)	−3.41 (−4.98–−2.05)	0.38 (−0.28–0.89)	0.78 (−0.94–2.09)	1.20 (−1.92–3.55)
Sleep to LPA	−0.87 (−1.30–−0.50)	−1.76 (−2.66–−0.97)	−2.65 (−4.07–−1.42)	0.98 (0.72–1.18)	1.98 (1.15–2.59)	2.96 (1.32–4.20)
Sleep to MVPA	−0.72 (−1.45–−0.08)	−1.44 (−2.96–−0.12)	−2.18 (−4.51–−0.14)	0.75 (0.06–1.27)	1.50 (−0.08–2.69)	2.24 (−0.39–4.23)
SED to Sleep	1.14 (1.55–0.78)	2.28 (3.02–1.62)	3.42 (4.43 –2.54)	−0.37 (−0.15–−0.53)	−0.72 (−0.76–−0.69)	−1.06 (−1.83–−0.48)
SED to LPA	0.28 (0.31–0.25)	0.57 (0.62–0.53)	0.89 (0.94–0.84)	0.60 (0.86–0.41)	1.20 (1.53–0.95)	1.80 (2.02–1.12)
SED to MVPA	0.43 (0.17–0.66)	0.89 (0.32 –1.38)	1.37 (0.49–2.13)	0.37 (0.18–0.52)	0.73 (1.08–0.52)	1.08 (0.11–1.81)
LPA to Sleep	0.86 (1.24–0.53)	1.72 (2.42–1.11)	2.56 (3.52–1.72)	−0.98 (−1.02–−0.95)	−1.96 (−2.34–−1.67)	−2.94 (−3.95–−2.17)
LPA to SED	−0.26 (−0.30–−0.22)	−0.50 (−0.60–−0.42)	−0.73 (−0.90–−0.58)	−0.61 (−1.02–−0.29)	−1.22 (−2.19–−0.48)	−1.84 (−3.50–−0.58)
LPA to MVPA	0.16 (−0.16 –0.44)	0.33 (−0.37–0.94)	0.51 (−0.59–1.46)	−0.24 (−0.76–0.15)	−0.51 (−1.65–0.36)	−0.80 (−2.66–0.61)
MVPA to Sleep	0.72 (1.30–0.21)	1.44 (2.27–0.71)	2.17 (2.60–1.80)	−0.76 (−0.32–−1.10)	−1.53 (−0.94–−1.98)	−2.33 (−1.92–−2.63)
MVPA to SED	−0.41 (−0.23–−0.56)	−0.78 (−0.64–−0.91)	−1.12 (−1.46–−0.82)	−0.39 (−0.34–−0.43)	−0.80 (−0.85–−0.76)	−1.23 (−1.55–−0.98)
MVPA to LPA	−0.14 (0.05–−0.31)	−0.27 (−0.14–−0.38)	−0.36 (−0.81–0.04)	0.21 (0.60–−0.08)	0.39 (1.00–−0.07)	0.53 (1.16–0.06)

SED: sedentary time, LPA: light physical activity, MVPA: moderate-to-vigorous physical activity, FEV_1%predicted_: forced expiratory volume in the first second predicted.

**Table 5:** Percentage change values of FEV_1%predicted_ when reallocating 30 min amongst different movement behaviours, stratified by sex, genotype and age in children and adults.

	Children				Adults			
Reallocation	Homozygous		Heterozygous		Homozygous		Heterozygous	
	Girls (27)	Boys (22)	Girls (14)	Boys (23)	Females (9)	Males (11)	Females (12)	Males (11)
Sleep to SED	−2.44 (−3.56–−1.76)	−2.46 (−3.38–−1.71)	−3.04 (−3.45–−1.69)	−2.38 (−3.27–−1.65)	−3.58 (−4.71–−2.64)	−3.24 (−4.49–−2.53)	−3.40 (−4.50–−2.50)	−3.24 (−4.35–0.97)
Sleep to LPA	−1.59 (−2.47–−1.10)	−1.64 (−2.08–−1.18)	−1.29 (−2.52–−0.95)	−1.59 (−2.26–−1.03)	−2.39 (−3.28–−1.61)	−2.10 (−2.80–−1.69)	−2.27 (−3.33–−1.39)	−2.16 (−3.08–1.21)
Sleep to MVPA	−1.11 (−2.88–0.33)	−1.07 (−2.47–3.32)	−1.66 (−2.65–0.11)	−1.03 (−2.26–−0.10)	−1.56 (−2.46–−0.73)	−1.98 (−1.83–−1.69)	−1.48 (−2.15–−0.83)	−1.41 (−1.63–2.36)
SED to Sleep	2.56 (3.01–2.09)	2.46 (2.86–2.14)	1.87 (3.05–2.01)	2.38 (2.76–1.96)	3.58 (4.30–3.22)	3.40 (3.69–3.09)	3.24 (4.11–3.05)	3.24 (3.62–6.52)
SED to LPA	0.98 (1.10–0.77)	0.94 (1.30–0.64)	0.35 (0.93–0.84)	0.91 (1.51–0.21)	1.37 (1.43–1.46)	1.29 (1.60–1.13)	1.13 (1.37–1.39)	1.23 (1.27–4.72)
SED to MVPA	0.73 (−0.41–1.54)	0.59 (−0.26–1.28)	0.12 (−0.27–1.27)	0.57 (0–1.03)	0.85 (0.82–1.02)	0.97 (1.03–0.70)	0.81 (1.17–0.83)	0.77 (1.27–4.02)
LPA to Sleep	1.71 (1.92–1.43)	1.53 (1.56–2.50)	0.94 (1.99–1.16)	1.47 (2.89–1.24)	2.22 (2.66–2.05)	2.10 (2.17–2.11)	2.11 (2.94–1.66)	2.00 (2.36–5.41)
LPA to SED	−0.73 (−1.23–−0.55)	−0.82 (−1.30–−0.43)	−1.40 (−0.93–−0.74)	−0.79 (−1.13–−0.62)	−1.19 (−1.43–0.88)	−1.13 (−1.64–−0.70)	−1.13 (−0.98–−0.97)	−1.08 (−1.27–2.64)
LPA to MVPA	1.59 (0.68–2.20)	1.53 (0.78–2.03)	0.94 (0.80–1.90)	1.36 (1.01–1.75)	2.05 (2.25–2.05)	2.10 (2.55–1.69)	1.94 (2.54–1.80)	2.00 (2.54–4.99)
MVPA to Sleep	0.98 (2.33–−0.33)	0.82 (1.69–0.11)	0.23 (1.99–−0.11)	0.79 (1.51–0.21)	1.19 (1.02–1.61)	1.29 (0.08–1.97)	1.13 (0.78–1.66)	1.08 (0–5.55)
MVPA to SED	−1.47 (−0.68–−2.31)	−1.53 (−1.04–−1.82)	−2.11 (−0.93–−2.01)	−1.47 (−1.26–−1.65)	−2.22 (−3.07–−1.46)	−1.94 (−3.54–−98)	−2.11 (−3.13–−1.25)	−2.00 (−3.62–2.77)
MVPA to LPA	−0.005 (0.41–−1.54)	−0.006 (0.13–−1.28)	−0.011 (−0.13–−1.16)	−0.006 (−0.38–−0.93)	−0.006 (−1.84–−0.29)	−0.005 (−2.21–0)	−0.006 (−1.96–0)	−0.006 (−2.54–3.74)

SED: sedentary time, LPA: light physical activity, MVPA: moderate-to-vigorous physical activity, and FEV_1_%_predicted_: forced expiratory volume in the first second predicted.

## Data Availability

The datasets generated and/or analysed during the current study are not publicly available due to GDPR regulations and to protect individual privacy but are available from the corresponding author on reasonable request.
